# CARNOY’S SOLUTION INCREASES LYMPH NODES COUNT IN COLON CANCER SPECIMENS WHEN COMPARED TO FORMALIN FIXATION: A RANDOMIZED TRIAL

**DOI:** 10.1590/0102-672020210002e1656

**Published:** 2022-06-17

**Authors:** André Roncon DIAS, Marina Alessandra PEREIRA, Evandro Sobroza MELLO, Ivan CECCONELLO, Ulysses RIBEIRO-JR, Sergio Carlos NAHAS

**Affiliations:** 1 Gastroenterology, Cancer Institute, Hospital das Clínicas, Faculty of Medicine, Universidade de São Paulo, São Paulo, SP, Brazil;; 2 Pathology, Cancer Institute, Hospital das Clínicas, Faculty of Medicine, Universidade de São Paulo, São Paulo, SP, Brazil

**Keywords:** Pathology, surgical, Colorectal neoplasms, Lymph Nodes, Neoplasm staging, Formaldehyde, Patologia cirúrgica, Neoplasias colorretais, Linfonodos, Estadiamento de neoplasias, Formaldeído

## Abstract

At least 12 lymph nodes (LNs) should be examined following surgical resection of colon cancer. As it is difficult to find small LNs, fat clearing fixatives have been proposed, but there is no consensus about the best option.

**AIM:**

The objective of this study was to verify if Carnoy’s solution (CS) increases the LN count in left colon cancer specimens.

**METHODS:**

A prospective randomized trial (clinicaltrials.gov registration: NCT02629315) with 60 patients with left colon adenocarcinoma who underwent rectosigmoidectomy. Specimens were randomized for fixation with CS or 10% neutral buffered formalin (NBF). After dissection, the pericolic fat from the NBF group was immersed in CS and re-dissected (Revision). The primary endpoint was the total number of LNs retrieved.

**RESULTS:**

Mean LN count was 36.6 and 26.8 for CS and NBF groups, respectively (p=0.004). The number of cases with <12 LNs was 0 (CS) and 3 (NBF, p=0.237). The duration of dissection was similar. LNs were retrieved in all cases during the revision (mean: 19, range: 4-37), accounting for nearly 40% of the LNs of this arm of the study. After the revision, no case was found in the NBF arm with <12 LNs. Two patients had metastatic LNs during the revision (no upstaging occurred).

**CONCLUSION:**

Compared to NBF, CS increases LN count in colon cancer specimens. After conventional pathologic analysis, fixing the pericolic fat with CS and performing a second dissection substantially increased the number of LNs.

## RESUMO - RACIONAL

Pelo menos 12 linfonodos (LNs) devem ser examinados após a ressecção cirúrgica do câncer de cólon. Como é difícil encontrar LNs pequenos, fixadores de clareadores de gordura foram propostos, mas não há consenso sobre a melhor opção.

**OBJETIVO:**

Verificar se a solução de Carnoy (SC) aumenta o número de LNs obtidos em espécimes de câncer de cólon esquerdo.

**MÉTODOS:**

Ensaio prospectivo randomizado (clinictrials.gov: NCT02629315) com 60 pacientes com adenocarcinoma de cólon esquerdo submetidos à retossigmoidectomia. As amostras foram randomizadas para fixação com SC ou formalina tamponada neutra a 10% (NBF). Após a dissecção, a gordura pericólica do grupo NBF foi imersa em SC e redissecada (Revisão). O *endpoint* primário foi o número total de LNs recuperados.

**RESULTADOS:**

O número médio de LNs foi de 36,6 e 26,8 para os grupos CS e NBF, respectivamente (p=0,004). O número de casos com <12 LNs foi 0 (CS) e 3 (NBF, p=0,237). A duração da dissecção foi semelhante. LNs foram recuperados em todos os casos durante a revisão (média de 19, intervalo: 4-37), representando quase 40% dos LNs deste braço do estudo. Após a revisão, nenhum caso no braço NBF permaneceu com <12 LNs. Dois pacientes tiveram LNs metastáticos encontrados durante a revisão (não ocorreu *upstaging*).

**CONCLUSÃO:**

Em comparação com NBF, a SC aumenta a contagem de LNs em espécimes de câncer de cólon. Após a análise patológica convencional, a fixação da gordura pericólica com SC e a realização de uma segunda dissecção aumentaram o número de LNs.

## INTRODUCTION

Colorectal cancer is one of the most diagnosed cancers worldwide^2^. Surgical resection is the main therapeutic option, and the analysis of at least 12 lymph nodes (LNs) is required to determine staging and prognosis^1^ ^,^ ^3^ ^,^ ^13^. The detection of LN in colorectal specimens is laborious and time-consuming. To facilitate and improve the detection of LN, tissue fixatives with fat clearing ability have been proposed, but there is no consensus about the best option or their clinical value^7^ ^,^ ^9^ ^,^ ^10^ ^,^ ^11^ ^,^ ^12^ ^,^ ^15^. In a randomized trial, Carnoy’s solution (CS, 60% ethanol + 30% chloroform + 10% glacial acetic acid) substantially increased LN count and improved staging accuracy in rectal cancer specimens after chemoradiation therapy when compared to 10% neutral buffered formalin (NBF)^4^. Other randomized trials validated CS in specimens with gastric cancer ^5^. LN retrieval is troublesome in rectal cancer following chemoradiation therapy, and in gastric cancer, the required number of LNs for adequate staging is high^16^.

But what about using a solution that reveals LNs for colon cancer specimens in a service with already high LN count? The present trial was proposed to address this issue.

## METHODS

The study was set in a reference cancer center in São Paulo, Brazil, between March 2012 and September 2013. It was approved by our institutional ethics committee (04248912.7.0000.0065) and registered at clinicaltrials.gov (NCT02629315). Informed consent was obtained before surgery during outpatient evaluation. Five board-certified surgeons performed the procedures. One pathologist handled all included specimens.

### Study design

**Figure 1 -:**
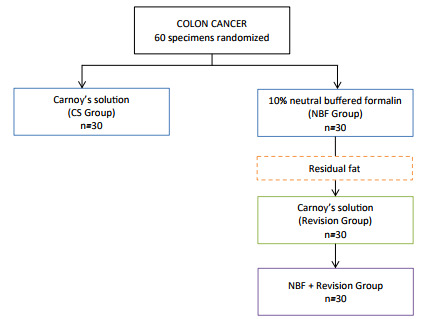
Study flowchart.

Sixty patients with left colon cancer who underwent rectosigmoidectomy had their surgical specimen randomly assigned for fixation with NBF (NBF group) or CS (CS group). The randomization ratio was 1:1 (Figure 1).

Specimens were fixed for at least 24 h. Pathological processing following the guidelines was presented elsewhere^14^. The pericolic fat from the mesocolon was weighted (grams) and measured (centimeters) in three axes to estimate volume. LNs were manually dissected (duration recorded in minutes) and counted two times to avoid errors. After dissection, the residual fat from the NBF group was immersed in CS for another 24 h and dissected again, in order to search for missed LNs (Revision). Final data from this arm of the study were classified as the NBF+Revision group.

### Participants

All patients with histologically confirmed left colon adenocarcinoma scheduled for rectosigmoidectomy were considered fit for inclusion in the study. The inclusion criteria were ligation of the inferior mesenteric artery at its origin, ligation of the inferior mesenteric vein close to the pancreatic inferior border, the release of the splenic angle of the colon, and section at the level of the rectum (sacral promontory).

Exclusion criteria were previous colonic surgical procedures, previous abdominal radiation therapy, multivisceral resections, a previous episode of diverticulitis, and obstructive lesions operated in an urgency setting.

As the specimen was randomized once all the above criteria were fulfilled, there was no loss and all cases were analyzed.

To help estimate sample size and verify the validity of the study protocol, data from 42 consecutive patients operated before the study and, under the same inclusion/exclusion criteria, were retrospectively collected. All retrospective cases were fixed in NBF and manually dissected. An exploratory analysis of the 5-year survival of both arms and retrospective cases was performed for external validity.

### Outcomes

The primary outcome was the total number of LNs retrieved. Secondary outcomes were the number of cases with <12 LNs and the duration of dissection. Variables that might correlate with LN count were also analyzed.

### Sample size

The main endpoint was the mean number of LNs harvested per patient. We expected to achieve a lower number of cases with <28 LNs using CS. This cutoff was based on the mean LN count for cancer rectosigmoidectomy in the 42 retrospective cases.

A pilot study was conducted with 10 cases in each arm, and its analysis showed that 30% of the cases in the NBF group had <28 LNs (none in CS). A sample size of 23 specimens per arm would be necessary to ensure statistical power of 80% (B error of 0.2) at a two-sided alpha of 5% (alpha error of 0.05). The sample was rounded to 30 cases in each arm.

### Randomization sequence

SAS Enterprise Guide version 4.3 was used for randomization. The data were computer-generated after each surgery by the statistician, once all eligibility criteria were matched. Due to this reason, surgeons remained blind during the procedure, and no assigned case was lost. As the solutions have a characteristic odor, the pathologist was not blinded.

### Statistical analysis

SPSS for Windows, version 20.0 (SPSS Inc, Chicago, IL) was used for statistical analysis. Nominal variables were studied using the chi-square test and continuous ones using the t-test or Mann-Whitney U test. The numerical variables (pericolic fat volume and weight, surgical specimen size, and duration of dissection) were also categorized into two groups based on the median value. To measure the linear relationship between two variables, Pearson correlation coefficient (*r*) was used. Overall survival (OS) was calculated from the surgery date until the date of death and estimated with the Kaplan-Meier method; the log-rank test was used to evaluate the difference between survival curves. The tests were two-sided, and p<0.05 was considered significant.

## RESULTS

### Carnoy’s solution vs. neutral buffered formalin

Thirty patients were included in each arm (Figure 1). CS and NBF groups were similar concerning sex, age, body mass index (BMI), surgical access, and pTNM (Table 1). The pericolic fat weight was higher in the CS group (p=0.039), and the mean fat volume was 1,889 vs. 1,375 cm^3^ for CS and NBF, respectively (p=0.079). There was no difference in pT (p=0.284), pN (p=0.301), and pTNM (p=0.301) status between the groups.

**Table 1 -:** Results from the prospective analysis

Variables	CS	NBF	Revision	NBF + Revision	p^1 CS vs NBF^
	n=30 (%)	n=30 (%)			p^2 CS vs NBF+Revision^
Sex					p^1^ =1
Female	18 (60)	18 (60)	-	-	
Male	12 (40)	12 (40)	-	-	
Age (years)					p^1^ =0.721
Mean (SD)	66.3 (10.7)	65.2 (12.3)	-	-	
BMI (kg/m²)					p^1^ =0.571
Mean (SD)	27.2 (4.8)	26.5 (4.4)	-	-	
Pericolic fat volume (cm^3^)					p^1^ =0.079
Mean (SD)	1889.4 (1316.3)	1375.3 (866.2)	-	-	
Median (range)	1735.3 (258-6000)	1284 (195-4064)	-	-	
Pericolic fat weight kg)					**p^1^ =0.039**
Mean (SD)	469.6 (270.8)	335.8 (216.4)	-	-	
Median	411.5 (143-1310)	299.9 (62.2-949.2)	-	-	
Surgical access					p^1^=0.273
Laparoscopic	12 (40)	8 (26.7)	-	-	
Open	18 (60)	22 (73.3)	-	-	
Dissection duration (min)					p^1^=0.957
Mean (SD)	46.2 (13.9)	46.0 (9.4)	29.6 (8.8)	75.6 (16.5)	**p^2^ =0.001**
Median	45 (20-100)	46 (25-65)	29 (20-50)	75 (45-110)	
Number of LNs					**p^1^=0.004**
Mean (SD)	36.6 (13.7)	26.8 (11.8)	19.0 (9.5)	45.8 (14.4)	**p^2^=0.014**
Median	35 (16-69)	28 (3-49)	19.5 (4-37)	41 (17-74)	
Cases with <12 LNs					p^1^ =0.237
No	30 (100)	27 (90)	-	30 (100)	p^2^=1.0
Yes	0 (0)	3 (10)	-	0 (0)	
Lymphatic invasion					p^1^ =0.390
No	20 (66.7)	23 (76.7)	-	-	
Yes	10 (33.3)	7 (23.3)	-	-	
Perineural invasion					p^1^ =0.390
No	20 (66.7)	23 (76.7)	-	-	
Yes	10 (33.3)	7 (23.3)	-	-	
Venous invasion					p^1^ =1.0
No	29 (96.7)	29 (96.7)	-	-	
Yes	1 (3.3)	1 (3.3)	-	-	
pT status					p^1^ =0.284
pT1/T2	13 (43.3)	9 (30)	-	-	
pT3/T4	17 (56.7)	21 (70)	-	-	
pN status					p^1^ =0.301
pN0	18 (60)	14 (46.7)	-	-	
pN1	12 (40)	16 (53.3)	-	-	
pTNM					p^1^ =0.301
0-II	14 (46.7)	18 (60)	-	-	
III-IV	16 (53.3)	12 (40)	-	-	
Surgical margins					p^1^ =1.0
Free	30 (100)	29 (96.7)	-	-	
Affected	0 (0)	1 (3.3)	-	-	

SD, standard deviation; BMI, body mass index; LN, lymph node; p-values in bold are statistically significant.

The duration of dissection was similar between the groups, and the mean number of retrieved LNs was 36.6 and 26.8 for CS and NBF groups, respectively (p=0.004). The number of cases retrieved with <12 LNs was 0 (CS) and 3 (NBF) (p=0.237, Table 1).

### Revision

**Figure 2 -:**
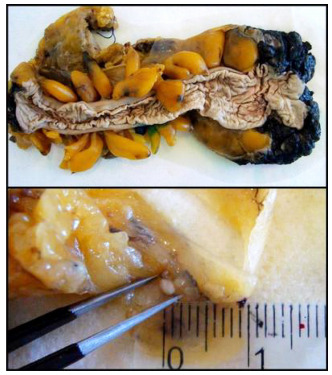
Surgical specimen and a millimeter lymph node fixed in Carnoy’s solution (CS group)

The mean dissection time in the Revision group was 29.6 min (range 20-50); LNs were retrieved in all cases (mean of 19 nodes per patient, range: 4-37). The size of LNs ranged from 1 to 3 mm (Figure 2). After the revision, there was no case in the NBF arm with <12 LNs. Two patients had metastatic LNs found in the Revision group, but their N status remained unchanged (Table 2).

**Table 2 -:** Cases in the NBF group with remarkable changes due to the Revision

NBF cases with ≥12 LNs due to the Revision group					
Case	LNs NBF	LNs Revision	LNs NBF+Revision	pTNM	Final Stage
1	1+/10	0/26	1+/36	pT2 N1 M0	IIIA
2	1+/3	0/14	1+/17	pT3 N1 M0	IIIB
3	0/11	0/30	0/41	pT1 N0 M0	I
**Cases with positive LNs in the Revision group**					
**Case**	**LNs NBF**	**LNs Revision**	**LNs NBF+Revision**	**pTNM**	**Final Stage**
4	5+/46	1+/23	6+/69	pT3 N2 M1	IV
5	9+/34	1+/4	10+/38	pT3 N2 M1	IV

LN, lymph node; NBF, 10% neutral buffered formalin

### Carnoy’s solution vs. neutral buffered formalin + Revision

The mean duration of dissection was longer and LN count was higher in the NBF+Revision group than in the CS group (75.6 vs. 46.2 min and 45.8 vs. 36.6) (Table 1).

### Clinicopathological variables and LN count

Sex (male vs. female, p=0.458), age (<65 vs. ≥65, p=0.867), and surgical access (open vs. laparoscopic, p=0.458) did not correlate with LN count. BMI ≥25 (p=0.020), high pericolic fat volume (≥1632.4 cm^3^, p<0.001), and size of surgical specimen (≥26 cm, p=0.032) were all associated with reduced LN yield (Table 3).

**Table 3 -:** Relationship between LN count and clinicopathological variables

Variables	n	%	Mean no. of LNs	p
Sex				0.458
Female	36	60.0	42.4	
Male	24	40.0	39.5	
Age (years)				0.867
<65	28	46.7	40.9	
≥65	32	53.3	41.5	
BMI (kg/m²)				**0.020**
<25	22	36.7	46.9	
≥25	38	63.3	37.9	
Pericolic fat volume (cm^3^)*				**<0.001**
<1632.4	35	58.3	47.3	
≥1632.4	25	41.7	32.7	
Pericolic fat weight (kg)*				0.088
<366.4	30	50.0	44.4	
≥366.4	30	50.0	38.0	
Surgical specimen size (cm)*				**0.032**
<26	32	53.3	45.0	
≥26	28	46.7	36.9	
Surgical access				0.153
Laparoscopic	20	33.3	45.1	
Open	40	66.7	39.3	
Dissection duration (min)				0.092
<57.5	30	50,0	38	
≥57.5	30	50,0	44.4	
Lymphatic invasion				0.402
No	43	71.7	42.2	
Yes	17	28.3	38.7	
Perineural invasion				0.960
No	43	71.7	41.1	
Yes	17	28.3	41.3	
Venous Invasion				0.908
No	58	96.7	41.2	
Yes	2	3.3	40.0	
pT				0.400
pT1/T2	22	36.7	43.3	
pT3/T4	38	63.3	40.0	
pN status				0.490
pN0	32	53.3	42.4	
pN1	28	46.7	39.8	
pTNM				0.490
0-I-II	32	53.3	42.4	
III-IV	28	46.7	39.8	

BMI, body mass index; LN, lymph node; p-values in bold are statistically significant; *cutoff values were determined according to the median values.

**Figure 3 -:**
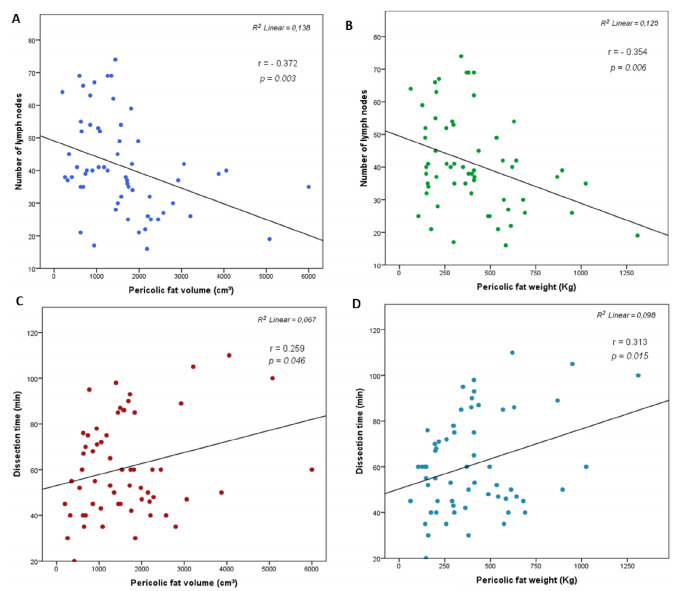
Relationship between the number on lymph node retrieval with (a) the volume and (b) weight of the pericolic fat; and between the duration of the dissection with (c) the pericolic fat volume and (d) weight (r=Pearson correlation coefficient).

In the correlation test, the number of LNs obtained were negatively correlated with the volume and weight of the pericolic fat (r=0.372, p=0.003; and r=0.354, p=0.006, respectively). Conversely, the meantime of dissection was positively correlated with the pericolic fat volume and weight (r=0.259, p=0.046; and r=0.313, p=0.015, respectively, Figure 3). There was no association between the number of LNs and the duration of the dissection (r=0.106, p=0.418).

### Retrospective group

Forty-two cases from the retrospective group were equivalent to the 30 cases from the NBF group in terms of sex (p=0.102), age (p=0.404), BMI (p=0.976), surgical access (p=0.870), pTNM (p=0.561), and LN count (mean 28.5 vs. 26.8, p=0.644).

### Survival outcomes: 5-year survival

An exploratory analysis of the survival was performed. Both CS and NBF+Revision groups had an equivalent 5-year OS (81.5% and 80%, p=0.894). The entire prospective cohort had a similar 5-year OS compared with the cases in the retrospective group (80.8% vs. 75.7%, respectively, p=0.665).

## DISCUSSION

During the pathological evaluation of colorectal cancer specimens, all removed LNs should be examined to eliminate the risk of understaging of the patient. However, this is not an easy task, and finding small LNs may be challenging. Fat clearing solutions have been proposed to facilitate the detection of LNs^7^ ^,^ ^10^ ^,^ ^11^ ^,^ ^12^. A clear benefit is seen when LN count is low^11^ ^,^ ^12^, but apparently the impact is reduced or absent if the LN yield is high^7^ ^,^ ^10^. CS is a validated and inexpensive tissue fixative that improves detection and staging accuracy of LN in rectal cancer following chemotherapy^14^. The present study was designed to verify whether CS is capable of increasing the LN count in left colon cancer specimens in an institution with already high number of LNs with conventional pathological analysis.

LN yield was significantly higher in the CS group than in the NBF group. CS allowed an increase of 36.6% (26.8 vs. 36.6) in the number of LNs retrieved. The NBF group had more cases with <12 LNs (3 vs. 0), but this was not significant (a larger sample is necessary to test this hypothesis). The duration of dissection was similar, but the perivisceral fat was more in the CS group than in the NBF group and may have influenced this result.

Revising the perivisceral fat from the NBF group (CS immersion followed by a new dissection) was time-consuming. Almost 40% of the total number of LNs in the NBF arm of the study was found during the Revision, and a higher LN count was obtained compared with the CS group. Again, attention should be drawn to the fact that the CS group had heavier perivisceral fat, a characteristic that was correlated with a reduced LN count. This and the fact that the second dissection of nearly 30 min was performed may explain why the NBF+Revision group had a higher LN yield (compared with the CS). Revision also allowed the three patients from the NBF group with <12 LNs to rise above this cutoff point. There was no upstaging after revision: two patients had metastatic LNs being missed after NBF fixation, but their pTNM status remained unchanged. Small metastatic LNs may indeed be missed by conventional analysis, and a larger sample is desirable to understand how this is translated into clinical practice and survival^8^.

As pericolic fat weight and volume, and BMI increased, retrieval of LN diminished. This is probably due to the difficulty in identifying small LNs amid all the fat tissue. It has been reported that an increase of BMI by 1.0 decreases LN count by 3.1%^6^.

The present study has the limitations of being unicentric, having a relatively small number of cases included, and the fact that a second dissection was not performed in the CS group. Many measures were taken to ensure the internal validity of the study (i.e., randomization, specialized surgeons and pathologists, and strict inclusion criteria), including only standard procedures. The high LN count and the absence of LN >3 mm in the Revision group attest the quality of the first dissection in the NBF arm. The 5-year survival was analyzed to verify if groups and treatments were comparable in the long-term, and to observe the external validity of the study (it was comparable to the survival of retrospective cases). In addition, LN count was equivalent in both the NBF group and the retrospective cases.

## CONCLUSION

Compared to NBF, CS increases the LN count in colon cancer specimens. The reduction in the number of cases <12 LNs should be verified in a larger population. The duration of the dissection was similar among solutions. After conventional pathological analysis, fixing the pericolic fat with CS and performing the second dissection substantially increased the number of LNs.
